# The Invasion and Metastasis of Colon Adenocarcinoma (COAD) Induced by SALL4

**DOI:** 10.1155/2022/9385820

**Published:** 2022-06-01

**Authors:** Wenjuan Zhang, Yan Hu, Wenbing Zhang, Ke Yi, Xiaohui Xu, Zhihua Chen

**Affiliations:** ^1^Department of Anesthesiology, QingPu Branch of Zhongshan Hospital Affiliated to Fudan University, Shanghai, China; ^2^The First People's Hospital of Taicang City, Taicang Affiliated Hospital of Soochow University, Suzhou, Jiangsu, China; ^3^Suzhou Medical College of Soochow University, Suzhou, Jiangsu., China

## Abstract

**Objective:**

The development and progression of many cancers may be related to SALL4, the role and molecular mechanism of which are unclear in colon adenocarcinoma (COAD).

**Methods:**

The SALL4 expression in adjacent normal mucosa tissues and carcinoma tissues of patients with COAD was detected through bioinformatic analysis based on TCGA database and immunohistochemistry. Single-cell analysis showed that the expression of SALL4 in normal tissue was noticeably low. GSEA analysis suggested that the SALL4 upregulated the GO and pathway of growth and cancer development and downregulated metabolization pathway. The relationship between lymph node metastasis, histological grading, clinical staging, and the expression of SALL4 in carcinoma tissues was analyzed. The upregulated or downregulated SALL4 expression of COAD cell lines was established. The influence of SALL4 on COAD cells invasion and proliferation was detected using plate cloning assay and Transwell. The expressions of EMT-related proteins E-cadherin, N-cadherin, vimentin, and Twist were detected by Western blot. The EMT phenotype was analyzed by immunofluorescence.

**Results:**

The study confirmed that the expression of SALL4 was upregulated in COAD and positively correlated with the degree of tumor differentiation, tumor staging, and metastasis. The overexpression of SALL4 was related to a poor prognosis, promoted the invasion and proliferation of colorectal cancer cells, and accelerated the occurrence of EMT, which was characterized by upregulation of Twist, vimentin, and N-cadherin expressions and downregulation of E-cadherin. The immunofluorescence staining confirmed the EMT phenotype. On the contrary, knocking out SALL4 gene reversed EMT, weakened cell proliferation and invasion, inhibited upregulation of Twist, vimentin, and N-cadherin expressions and downregulation of E-cadherin.

**Conclusion:**

To sum up, TNM grading, histological grading, and lymphatic metastasis were significantly correlated with SALL4 in tumor tissues. SALL4 played a vital role in tumor proliferation, invasion, and tumor EMT and may be a novel target for COAD.

## 1. Introduction

COAD accounts for about 10% of all cancer diagnoses and cancer-related deaths all over the world each year. It is the third most common cancer among male and the second most common cancer among female [1, 2]. Comprehensive treatment dominated by surgery can achieve better results in the managing early stage of COAD [3]. However, COAD treatment will become more challenging in the later stages, eventually developing resistance to most drugs and allowing COAD metastasis to be a leading factor resulting in cancer-related death [4]. The prognosis for COAD disease remains quite poor, with a 5-year survival rate of about 65.4% for stage III and 12.8% for stage IV [5]. The poor treatment results demonstrated the necessity to better study the underlying mechanisms leading to tumorigenesis and progression [6].

SALL4 is a transcription factor that belongs to the SALL gene family [7]. SALL4 helps maintain embryonic stem cells self-renewal or pluripotency [8, 9]. Its expression is gradually downregulated with development and is even silent in most adult tissues [10]. Recent findings have shown that SALL4 is reexpressed in cancer. The expression of SALL4 increases in acute myeloid leukemia (AML), liver cancer, breast cancer, endometrial cancer, lung cancer, and glioma [11–14]. Moreover, SALL4 has been found to act critically in tumor genesis, progression, and metastasis [15]. Specifically, SALL4 promotes cell migration, proliferation, drug resistance, and invasion by upregulating c-Myc in endometrial cancer [16]. SALL4 induces EMT by activating TGF-*β*/SMAD signaling pathway and promotes gastric cancer metastasis [17]. Due to its roles in various carcinogenic processes, SALL4 becomes a new biomarker for tumor diagnosis and treatment [18]. According to previous reports, SALL4 is high-expressed in the serum and tissues of COAD, and its expression level in serum and tissues is closely related to lymph node metastasis, differentiation degree, and Dukes staging. Higher SALL4 level in serum is positively associated with lower survival in patients with COAD. In other studies, real-time polymerase chain reaction (PCR) was used to determine the expression of SALL4 in fresh and distant tumor tissues of colorectal specimens and the level of serum peripheral blood mononuclear cells, and similar results were obtained. However, the role of SALL4 in COAD metastasis and its molecular mechanism is still unclear.

Epithelial mesenchymal transformation (EMT) is the phenomenon in which epithelial cells transform into mesenchymal cells and obtain interstitial features under specific physiological and/or pathological cases. As cell polarity disappears and the close connection between cells and adhesion connection disappear, cells will obtain the invasive and migration ability and also create new extracellular matrix components and inhibit cell apoptosis. It is an important link of embryonic development, tissue organ damage repair and tumor metastasis [19]. Studies increasingly confirmed a strong relation of EMT to cancer progression and metastasis of many malignancies, including COAD. The overexpression of the transcription factors SNAIL, SLUG, TWIST1, TWIST2, ZEB1, ZEB2, PROX1, FOXC2, FOXQ1, FOXC1, and FOXM1 [20–22] related with EMT is correlated with COAD invasion, metastasis, and poor prognosis. However, whether SALL4 was associated with EMT-related factors and the precise effects on colon cancer cells and their internal mechanisms have not been fully explored. It was assumed that the biological behavior of COAD cells could be regulated by discovering new EMT-related signaling pathways, thereby influencing the invasion and metastasis of COAD and improving the prognosis of patients. In the study, the effects of SALL4 interference and overexpression on the growth, proliferation, and migration of COAD cells were investigated, and the correlation between SALL4 and EMT was explored.

According to our results, SALL4 had significant effects on colorectal proliferation and invasion at the cellular level, and it was significantly correlated with EMT. The role of SALL4 in promoting lymph node metastasis and late clinical stage might be partly related to the interaction of EMT, which might be a new mechanism underlying the carcinogenic effect of SALL4.

## 2. Materials and Methods

### 2.1. TCGA Dataset Analysis

Relevant clinicopathological information and the TCGA dataset were from a public database cBioportal (http://www.cbioportal.org/). Based on Sangerbox (http://sangerbox.com/), a comprehensive tool for bioinformatic analysis of *R*, we performed the SALL4 expression in normal tissues and in pan-cancerous tissues and conducted survival assessment based on the SALL4 expression. We also retrieved clinical information from 458 COAD patients for correlation analysis and performed Cox regression analysis for overall survival (OS). The prognostic value of COAD patients based on various clinicopathological features was analyzed using the Kaplan-Meier plotter.

### 2.2. Single-Cell Sequencing Analysis

Brush cells are a compilation of single-cell transcriptome data from the model organism Mus musculus, which contains nearly 100,000 cells from 20 tissues and organs. Tabula Muris (https://tabula-muris.ds.czbiohub.org/) was employed for determining the SALL4 expression in brush cells [23]. Enterocyte of epithelium of larger intestine, brush cell of epithelium proper of large intestine, epithelial cell of large intestine, large intestine goblet cell, and enteroendocrine cell were contained in large intestine tissue.

### 2.3. Gene Set Enrichment Analysis

Using normalized RNA-Seq data by TCGA, GSEA was performed. In GSEA version 3.0, the gene sets annotated with c5.all.v7.0.symbols.gmt and c2.cp.kegg.v7.0.symbols.gmt from the Molecular Signature Database (MSigDB) were selected [24–26]. The normalized enrichment score was determined with permutations number set at 1,000. The GO term, KEGG pathway, was conducted using GSEA to investigate potential biological function of TLCD1. Statistically significant was defined if enrichment results meeting a false discovery rate FDR *q* value <0.25 and anominal *P* value <0.05.

### 2.4. Patient and Tissue Samples

The Taicang Affiliated Hospital of Soochow University Human Research Ethics Committee approved this study (KY-2017-005). 80 cases of samples of paracancer tissues were collected from 2019-01-01 to 2020-10-01, and appropriate informed consent was given to the patients. On average, 71 patients who underwent surgery were 64.8 years old. No patients received neoadjuvant chemoradiotherapy preoperatively.

### 2.5. Immunohistochemistry

All the specimens were cut into 4-*μ*m slices and baked, then deparaffinized, and rehydrated. The specimens were incubated with 3% H_2_O_2_ for 10 min, then incubated in citric acid buffer, and boiled. At 4°C, the slices were incubated with rabbit anti-human SALL4 antibody (1 : 100; Abcam Inc.) overnight. After washing with phosphate buffered saline (PBS), the samples were treated with goat anti-rabbit secondary antibodies (1 : 100; Beijing Kangwei Century Biotechnology Co., Ltd.). Next, the specimens were incubated with streptavidin-horseradish peroxidase and DAB (Wuhan Bode Biological Engineering Co., Ltd.; Wuhan, China) drop visualization and then antistained with hematoxylin. The normal nonimmune serum was replaced with SALL4 antibody as a negative control. For each slice, 5 high-power fields were selected, and nucleus without chromogeny was considered to be negative (-); 25% ~50% of the chromogenic and staining particles in the nucleus was considered to be weak positive (+); 50% ~75% of the chromogenic and staining granules was considered to be moderately positive (++); >75% of the nuclei staining and dark staining granules was considered to be strongly positive (+++). Staining intensity was classified as 1 (weak), 0 (negative), 2 (moderate), and 3 (strong) to determine the SALL4 level. The degree of dyeing was characterized by 0 (0%), 1 (1% ~25%), 2 (26% ~50%), 3 (51% ~75%), and 4 (76% ~100%). The sum of intensity score and degree score was the final staining score, with a final staining score ≥ 4 presenting positive.

### 2.6. SALL4 Plasmid Construction and Cell Transfection

SALL4 gene knockout cell line was established by using SW480 COAD cell line with the high SALL4 expression. COAD SW480 cells were transfected with plasmids NC-oe, SALL4-oe, NC-sh, and SALL4-sh. In the plasmid transfection, the cells were grew to 70~80% confluence in culture plate with 6 holes and then transfected with shRNA-SALL plasmid (GCTAGACACATCCAAGAAAGGTTCAAGAGACCTTTCTTGGATGTGTCTAGCTTTTTT, sequencing primers: U6 promoter universal primers GGACTATCATATGCTTACCG) or shRNA-NC plasmid (insert pLent of nonsense sequence pLent-U6-GFP-Puro) (Weizhen Biological Technology Co., Ltd.). the overexpressed SALL4 (5′-end sequencing primers: CGCAAATGGGCGGTAGGCGTG; 3′-end sequencing primers: CCTCTACAAATGTGGTGGC), with pENTER no-load as the control) was adopted. After 48 h, the cells were collected for testing. The interference efficiency of SALL4 gene was verified by real-time fluorescence quantitative PCR and Western blot detection.

### 2.7. Western Blot

The cells were added into RIPA lysate cells, and we used Bradford assay to analyze the protein concentration, followed by SDS-polyacrylate gel electrophoresis. Next, the proteins were moved onto nitrocellulose membrane (NC membrane) using a protein transfer device. After the NC membrane was immersed in 5% skim milk and sealed overnight at 4°C, the membrane was then combined with the first anti-mouse and anti-human E-cadherin antibody (1 : 50; Abcam), N-cadherin antibody (1 : 1000; Abcam), rabbit anti-human vimentin antibody (1 : 1000; Abcam), rat anti-human twist antibody (1 : 1000; Abcam), sheep anti-rat AFP antibody, CK-19 antibody (1 : 200 dilution), and *β*-actin antibody (1 : 400) and then closed and incubated at 4°C overnight, followed by membrane washing using PBS-T buffer for 3 times and TBST washing. The membrane was coincubated with biotin-labeled secondary antibody (1 : 5000 dilution) for 1.5 h and washed with PBS-T buffer for 3 times. Finally, the target protein bands were colorized with DAB. The gray value of protein was detected with the gel imaging analysis system.

### 2.8. Plate Cloning Formation Experiment

Logarithmic phase of cells was washed with PBS, followed by cell digest using trypsin cell digestion solution. Then, the cells were beaten evenly and counted, inoculated in a 6-hole plate at a certain concentration and cultured in the medium containing 10% fetal bovine serum. Each group had 3 double holes and cultured for 14d. After washing with PBS twice, cell fixation with methanol was conducted for 15 min, followed by 30 min GIEMSA staining. Cell colonies with more than 50 cells were calculated. >50 cells/colony were considered as a colony.

### 2.9. Transwell Experiment

After collection, logarithmic phase of cells was suspended in serum-free medium. After counting, the concentration was changed to 1^∗^10^5^/mL. The lower chamber of a 24-hole Transwell plate (Corning Inc., USA) (the bottom of the 24-hole plate) was added with 600 *μ*l of medium with 20% serum. The upper chamber was added with 150 *μ*l cell suspension and cultured under 37°C with 5% CO_2_ for 24 h. After careful removal of the chamber, liquid in the upper one was aspirated, followed by methanol fixation for 30 min at room temperature. After the chamber removal, fixation solution was eliminated from upper chamber. Crystal violet dye was stained for 15~30 min at room temperature, following by washing and soaking in PBS for repeated times. After chamber removal, liquid was aspirated from the upper chamber, and the cells were carefully wiped from the membrane surface at the bottom of the upper chamber using wet cotton swabs. The bottom was kept upwards for airing and then moved to the slide, followed by neutral resin sealing. Five random fields under the microscope were taken for counting. In addition, three visual fields were selected for each Transwell chamber to be photographed, and the cells were counted under an inverted microscope.

A total of 1 × 104 cells were inoculated in the upper Boyden chamber and filtered with an 8 *μ*m pore diameter membrane. Then, 10%-FBS medium as chemical attractant was added into the lower chamber. The cells from the upper filter after 24 h were gently removed with a cotton swab. The cells moved to the filter membrane lower surface were fixed with 4% paraformaldehyde, followed by 10 min hematoxylin staining, and then washed for 3 times to remove hematoxylin. The filter of the membrane was dried with a hair dryer, and the migratory cells (randomly 10 200× fields per hole) were calculated. We operated three independent experiments. Data were displayed as mean ± S.E.M.

### 2.10. Immunofluorescence

After cell inoculation, COAD cells were performed with 4%-paraformaldehyde fixation for 15 min. Goat serum (SolarBio, China) sealed the slices after 30 min of infiltration with 0.25% Triton X-100 (Beyotime, China) at room temperature. Subsequently, they were treated with N-cadherin (1 : 100, 66219-1-Ig, Proteintech, China) overnight at 4°C. Cy3-conjugated IgG (1 : 400, A0521, Beyotime) or Cy3-conjugated IgG (1 : 400, A0516, Beyotime) of COAD cells was cultured with goat anti-mouse (1 : 400, A0521, Beyotime) or goat anti-rabbit Cy3 (1 : 400, A0516, Beyotime). Finally, the cells were restained with DAPI (Sigma, USA) before taking the images by a fluorescence microscope (Olympus, Japan).

### 2.11. Analysis and Validation of SALL4

To further confirm the important roles of hub genes in the pathogenesis and prognosis of COAD, we verified their expression and prognostic significance, according to the GEPIA database. GEPIA, an interactive web application tool for gene expression analysis, contains 8587 normal samples and 9736 tumors samples from the Genotype-Tissue Expression databases and The Cancer Genome Atlas (TCGA) databases [27]. Furthermore, RNA-sequencing expression profiles were downloaded from the TCGA dataset (https://portal.gdc.com). Spearman's correlation analysis was performed to describe the correlation between quantitative variables without a normal distribution. *P* values less than 0.05 were considered statistically significant.

### 2.12. Statistics Analysis

According to the International Union Against Cancer, the TNM Staging System Seventh Edition (AJCC) was used for COAD staging [28]. Consistency test was conducted through Kappa analysis, and difference of classification data was compared with *χ*^2^ test. Comparison between groups was conducted using *χ*^2^ segmentation. Correlation analysis of paired data was used to test the expression of each index.

## 3. Results

### 3.1. The Expression of SALL4 in COAD

**Figure 1:**
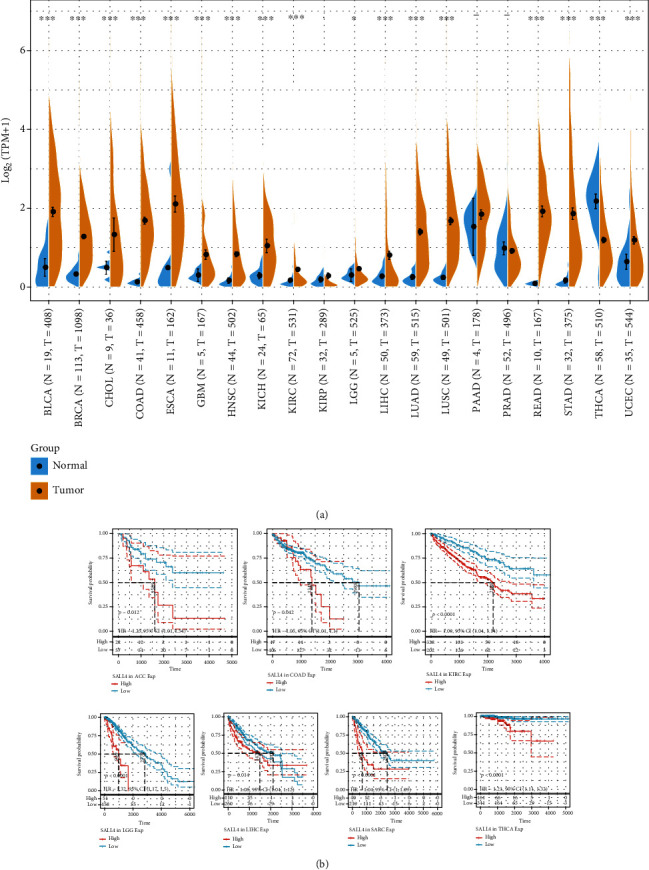
(a) Violin plot of pan-cancer analysis of the CALL4 expression based on the TCGA database. (b) Survival analysis of CALL4 in ACC, COAD, KIRC, LGG, LIHC, SARA, and THCA.

The Sangerbox database was applied to examine SALL4 in various cancers. As shown in Figure 1(a), the mRNA expression of SALL4 showed a significant upregulation in several common cancer datasets, including esophageal carcinoma (ESCA), breast invasive carcinoma (BRCA), colon adenocarcinoma/rectum carcinoma (COAD), bladder urothelial carcinoma (BLCA), colon adenocarcinoma (COAD), cholangiocarcinoma (CHOL), kidney renal clear cell carcinoma (KIRC), head and neck squamous cell carcinoma, kidney chromophobe (KICH), glioblastoma multiforme (GBM), lung adenocarcinoma (LUAD), liver hepatocellular carcinoma (LIHC), ovarian serous cystadenocarcinoma (OV), lung squamous cell carcinoma (LUSC), mesothelioma (MESO), lower-grade glioma (LGG), uterine corpus endometrial carcinoma (UCEC), prostate adenocarcinoma (PRAD), stomach adenocarcinoma (STAD), rectum adenocarcinoma (READ), thyroid carcinoma (THCA), and pancreatic adenocarcinoma (PAAD) [29].

### 3.2. The Prognostic Analysis of SALL4 Genes in COAD Patients

The TCGA-COAD was investigated using Sangerbox for determining how the SALL4 expression affected the prognosis of COAD patients.  Figure 1(b) shows that high-expressed SALL4 was associated with a poor prognosis of COAD (OS, *P* = 0.042).

### 3.3. SALL4 Expressed in Colon Cells

**Figure 2:**
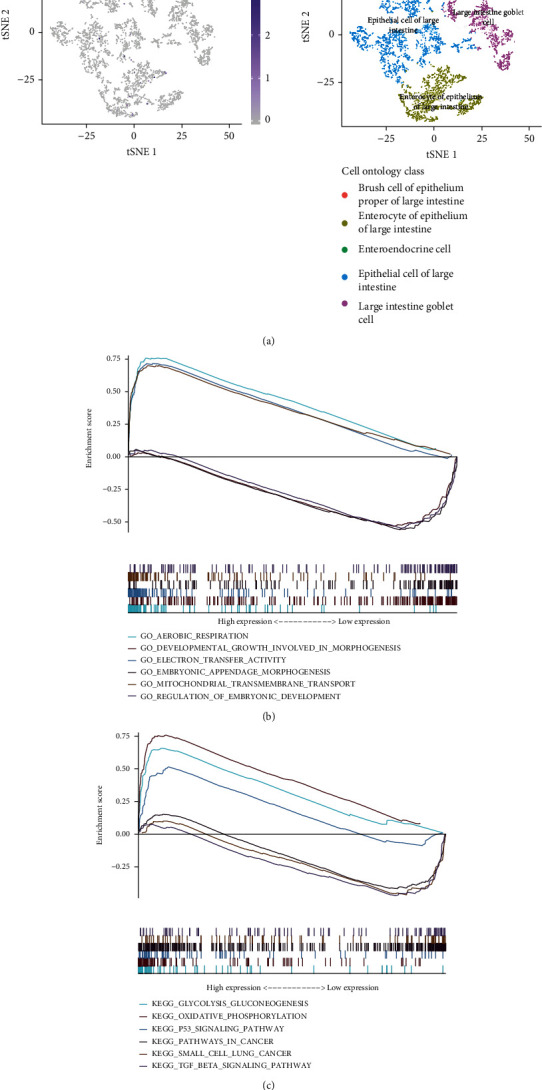
Interactive plots of (a) the SALL4 expression in colon cells and the categories of colon cells. (b) GO enrichment analysis of SALL4 in COAD via GSEA. (c) KEGG enrichment of SALL4 in COAD via GSEA.

Using single-cell sequencing of large intestine, we found that SALL4 expressed significantly low in enterocyte of epithelium of larger intestine, enteroendocrine cell, brush cell of epithelium proper of large intestine, large intestine goblet cell, and epithelial cell of large intestine included in large intestine tissue (Figure 2(a)).

### 3.4. GSEA Analysis Identified SALL4-Related Pathways in COAD

To determine the signal pathway related to the expression of SALL4 in COAD, gene set enrichment analysis (GSEA) was conducted between the expression data of low and high SALL4. GO annotation indicated that three positive pathways and five negative pathways related to the high expression of APCDD1L: GO_DEVELOPMENTAL_GROWTH_INVOLVED_IN_MORPHOGENESIS, GO_REGULATION_OF_EMBRYONIC_DEVELOPMENT, GO_EMBRYONIC_APPENDAGE_MORPHOGENESIS, GO_AEROBIC_RESPIRATION, GO_MITOCHONDRIAL_TRANSMEMBRANE_TRANSPORT, and GO_ELECTRON_TRANSFER_ACTIVITY. KEGG pathway revealed five positive pathways and five negative pathways: KEGG_SMALL_CELL_LUNG_CANCER, KEGG_PATHWAYS_IN_CANCER, KEGG_TGF_BETA_SIGNALING_PATHWAY, KEGG_P53_SIGNALING_PATHWAY, KEGG_GLYCOLYSIS_GLUCONEOGENESIS, and KEGG_OXIDATIVE_PHOSPHORYLATION (Figures 2(b) and 2(c)).

### 3.5. The Expression of SALL4 in COAD and Paracancerous Tissues

**Figure 3:**
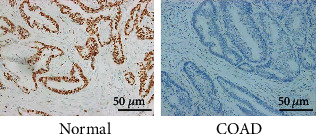
(a) The expression of SALL4 protein in colon tissues (×400) (left: SALL4 protein was positively expressed in COAD tissues; right: SALL4 protein was not expressed in paracancerous tissues).

The proteins positioning and expressing SALL4 protein are mainly localized in the nucleus. The expression rate of SALL4 in COAD tissues (73.75%, 59/80) was significantly higher than that in paracancerous tissues (7.5%, 6/80), with statistically significant differences (*χ*^2^ = 71.16, *P* < 0.001) (Table 1 and Figure 3(a)).

### 3.6. The Effects of SALL4 on Proliferation and Invasion of COAD

**Figure 4:**
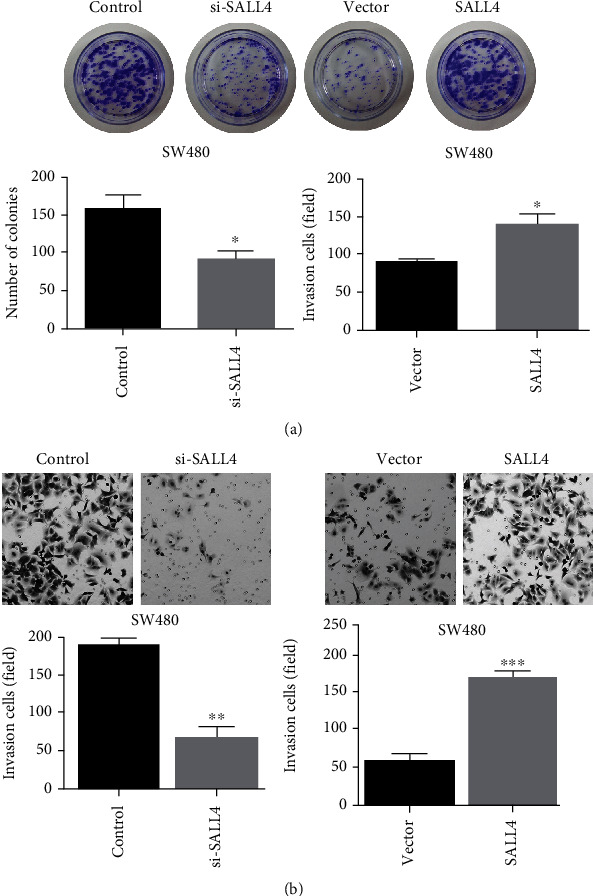
(a) SALL4 promoted the growth of SW480 cells, while the silenced SALL4 reduced the rate of cell clone formation in plate clone formation assay. (b) Compared with control cells, the migration rate of SW480 cells that expressed SALL4 significantly increased, while the migration rate of SW480 cells that silenced SALL4 decreased in Transwell invasion assay.

Overexpressed empty load group (NC-oe group), overexpressed group (SALL4-oe group), interfered empty load group (NC-sh group), and interfered expression group (SALL4-sh group) were set up for analyzing the effect of SALL4 on cell proliferation and invasion. As shown in Figure 4(a), the result of plate clone formation assay demonstrated that SALL4 promoted the growth of SW480 cells (*P* < 0.05 or 0.01). In contrast, the silenced SALL4 reduced the rate of cell clone formation. Transwell invasion assay was performed, as shown in Figure 4(b). In comparison with control cells, the migration rate of SW480 cells expressing SALL4 significantly increased, while that of SW480 cells with silenced SALL4 decreased (*P* < 0.01).

### 3.7. SALL4 Promoted EMT and Enhanced the Invasion of Colon Cancer Cells, while Silencing SALL4 Inhibited the EMT Phenotype and Reduced the Metastatic Potential of Colon Cancer Cells

**Figure 5:**
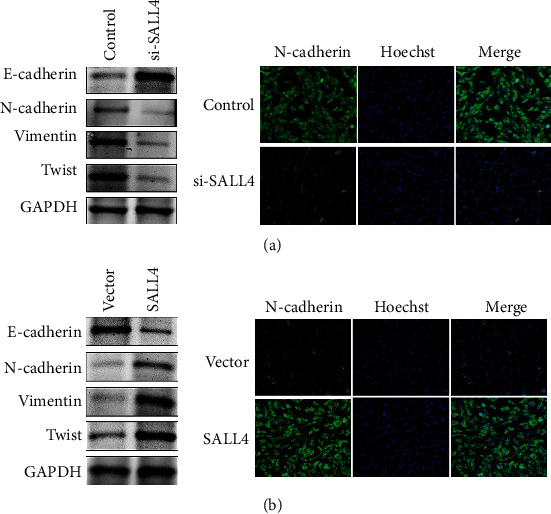
(a) The overexpression of SALL4 promoted EMT, which was manifested as downregulation of E-cadherin as well as the upregulation of N-cadherin, vimentin, and Twist. (b) On the contrary, knockout of SALL4 gene reversed EMT and inhibited EMT phenotype, which was manifested as upregulation of E-cadherin as well as the downregulation of N-cadherin, vimentin, and Twist expression.

To determine whether SALL4 induced EMT, the cells were examined by using epithelial marker E-cadherin and stromal markers N-cadherin and vimentin, as well as GAPDH and TWIST (two well-known EMT-related genes). As shown in Figure 5(a), the overexpression of SALL4 promoted EMT, which was manifested as downregulation of E-cadherin and the upregulation of N-cadherin, vimentin, and Twist. However, knockout of SALL4 gene reversed EMT and inhibited EMT phenotype, which was manifested as upregulation of E-cadherin and the downregulation of N-cadherin, vimentin, and Twist expression (Figure 5(b)). The EMT phenotype was confirmed by immunofluorescence staining.

### 3.8. Analysis and Validation of the SALL4 Expression in COAD

**Figure 6:**
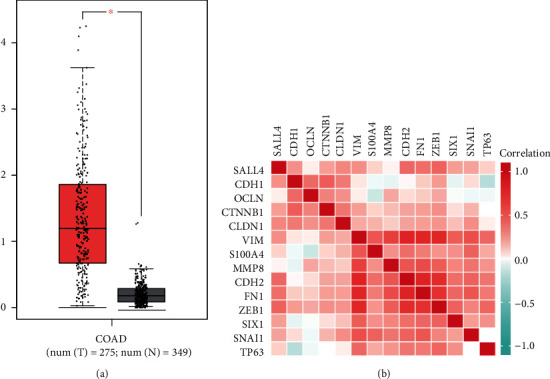
(a) The expression level of SALL4 between GEPIA and normal tissues in five datasets. (b) Heat map of the relationship between SALL4 and EMT-related genes.

The mining of the GEPIA database also demonstrated that SALL4 exhibited significant differences in the expression between tumor and normal tissues in COAD (Figure 6(a)). The heat map of the correlation between SALL4 and EMT-related genes was shown in Figure 6(b).

## 4. Discussion

SALL4 is a transcription factor that maintains the pluripotency and self-renewal of embryonic stem cells, and it is quite important in embryonic development [30]. As organs mature, the expression of SALL4 gradually decreases and is silenced in most adult tissues. However, it is reexpressed in germ cell tumors, leukemia, and malignancies [31, 32]. Recent studies have shown that SALL4 plays an important role in many solid tumors, including in esophageal cancer, cervical cancer, and gastric cancer. Moreover, in vivo and in vitro experiments have suggested the metastatic potential of SALL4 in tumors [33]. However, the functional role and molecular mechanism of SALL4 in COAD are still unclear. In this study, it was found that SALL4 was involved in the metastasis of COAD. Here in our study, in more than half of human COAD tissues, SALL4 showed a significant upregulation, and the current findings were consistent with recent studies analyzing the relationship of the SALL4 expression with COAD. It has been found that the expression of SALL4 in more than 87% of tumors detected was increased by two times in normal tissues [15]. Furthermore, TNM grade, histological grade, and lymphatic metastasis of COAD showed a close relationship with the level of the SALL4 expression, suggesting that SALL4 might play a vital role in the progression of COAD. To analyze how SALL4 functioned in COAD, the malignant potential of the increased and silenced SALL4 expression in COAD cells was demonstrated for the first time. SALL4 could regulate the migration, invasion, cloning, and proliferation of COAD cells. The results showed that the proliferation ability, invasion ability, migration ability, and clone formation ability of SW480 cells were reduced after the SALL4 silencing of COAD cells; however, the proliferation ability, invasion ability, migration ability, and clone formation ability of SW480 cells were enhanced after the overexpression of SALL4, further proving that SALL4 had a cancer-promoting effect.

EMT, which plays a significant role in tumor invasion and metastasis, embryonic development, is a main molecular mechanism enhancing metastasis and invasion in the process of promoting cancer development [34, 35]. It was found in our study that the migration and invasion of COAD cells were greatly limited by shRNA-induced SALL4 silencing. In addition, shRNA-induced SALL4 upregulated the expression of N-cadherin, vimentin, and Twist and downregulated E-cadherin through inhibiting the expression of N-cadherin, vimentin, and Twist. However, the overexpression of SALL4 accelerated the invasion and proliferation of colon cancer cells, which was manifested as downregulation of E-cadherin and upregulation of N-cadherin, vimentin, and Twist. The EMT phenotype was confirmed by immunofluorescence staining. Therefore, how to treat COAD through SALL4 gene and whether there was one of the key molecules between SALL4 and EMT should be studied in future.

## Figures and Tables

**Table 1:** The positive expression rate (cases) of SALL4 protein in different COAD tissue samples.

Clinicopathological factors		*n*	Negative expression	Positive expression	*χ* ^2^	*P* value
Age	>60	24	6	18	0.028	0.868
	≤60	56	15	41		
Gender	Male	54	16	38	0.98	0.322
	Female	26	5	21		
The degree of differentiation	High	22	2	20	4.615	0.032
	Medium-low	58	19	39		
Tumor size	<5 cm	52	15	37	0.517	0.472
	≥5 cm	28	6	22		
Clinical staging	I/II	26	3	23	4.306	0.038
	III/IV	54	18	36		
Lymph node metastasis	No	50	9	41	4.688	0.03
	Yes	30	12	18		
Depth of tumor invasion	T1 + T2	12	2	10	0.67	0.413
	T3 + T4	68	19	49		

## Data Availability

The data used to support the findings of this study are included within the article. More information can be accessed from correspondence authors.
